# Variables related to the quality of life of families that have a child with severe to profound intellectual disabilities: A systematic review

**DOI:** 10.1016/j.heliyon.2021.e07372

**Published:** 2021-06-24

**Authors:** N. Luitwieler, J. Luijkx, M. Salavati, C.P. Van der Schans, A.J. Van der Putten, A. Waninge

**Affiliations:** a Research Group Healthy Ageing, Allied Health Care and Nursing, Hanze University of Applied Sciences Groningen, Groningen, the Netherlands; b Department of Special Needs Education and Youth Care, University of Groningen, Groningen, the Netherlands; c Royal Dutch Visio, Center of Expertise for Blind and Visually Impaired People, Haren, the Netherlands

**Keywords:** Children with intellectual disabilities, Children with severe to profound intellectual disabilities, Intellectual disabilities, Severe to profound intellectual disabilities, Family quality of life, Variables

## Abstract

**Background:**

Family quality of life (FQoL) of families that have a child with severe to profound intellectual disabilities (SPID) is an important and emerging concept, however, related variables are inconclusive.

**Aim:**

To gain a better understanding of variables related to the FQoL of families that have a child with SPID, variables related to the FQoL of families that have a child with intellectual disabilities (ID) were systematically reviewed.

**Methods and procedures:**

A search strategy was performed in five databases. Critical appraisal tools were employed to evaluate the quality of both quantitative and qualitative studies. Data extraction and synthesis occurred to establish general study characteristics, variables, and theoretical concepts. Variables were categorised into four key concepts of the FQoL: systemic concepts, performance concepts, family-unit concepts and individual-member concepts.

**Outcomes and results:**

A total of 40 studies were retrieved with 98 variables. Quality scores ranged from 7 to 13 (quantitative) and 5 to 13 (qualitative) out of 13 and 14 points, respectively. Five out of the 40 studies (13%) focused on individuals with SPID. Variables related positively or negatively to the FQoL, and were categorised within systemic concepts (n = 3); performance concepts (n = 11); family-unit concepts (n = 26); and individual-member concepts (n = 58).

**Conclusions and implications:**

Several variables were found to be (inter)related to the FQoL of families that have a child with ID. A contrasting picture emerged regarding the impact of a disability in relation to transitional phases. However, studies which include families of children with SPID were minimal, therefore, it remained ambiguous to what extent the identified variables apply to these families.

**What this paper adds**

The Family Quality of Life (FQoL) concept offers insight into the functioning of families that have a child with intellectual disabilities (ID). This is the first systematic review performed that provides an overview of variables that are related positively or negatively to the FQoL in families that have a child with ID. Therefore, it contributes to the establishment of what is currently known about this topic and exposes any knowledge gaps, more specifically concerning families that have a child with severe to profound intellectual disabilities (SPID). This review also includes recommendations and therewith provides direction for further research.

## 1 Introduction

Family Quality of Life (FQoL) is a concept that helps to understand and improve the well-being of families raising a child with intellectual disabilities (ID). FQoL is defined by Zuna et al. (2010) as: “a dynamic sense of well-being of the family, collectively and subjectively defined and informed by its members, in which individual and family-level interact” (p. 262). The FQoL approach recognises the dynamics between family members and considers FQoL as the intersection where the individual perceived Quality of Life (QoL) meets the FQoL (Zuna et al., 2014). FQoL builds upon QoL research (e.g. Schalock et al., 2007), in which pre-existing QoL life domains (e.g. physical and material well-being, emotional well-being, social belonging, and community living) were elaborated with FQoL domains, such as daily family life, parenting, family interactions, and financial well-being (Poston et al., 2003). Over the last years, there has been an increased focus on FQoL research whereby studies focusing on FQoL, and family related concepts (e.g. family functioning, family well-being), have been performed from different theoretical perspectives. This trend relates to the progression towards a family-centered approach by professionals, that recognises the important role of family in a child's life (Alsem et al., 2013). Studies also focused on how families and the FQoL can be supported in the best possible way with a focus on strengths instead of just deficits (Brown and Brown, 2004; Schippers and Van Boheemen, 2009).

Raising a child with ID may have a major impact on the lives of parents, siblings, and the family (Trute and Hiebert-Murthpy, 2002). It has been realised that the support demands for children with severe to profound intellectual disabilities (SPID) who often have additional disabilities and complex needs are significantly negatively impacting family life (Hanson and Lynch, 2004; Lima-Rodríguez et al., 2017; Luijkx et al., 2017). Parenting these children is highly intensive and long lasting because, as they grow older, their support in basic needs only increases which makes them even more dependent on caregivers (Tadema and Vlaskamp, 2009). Unlike in the past when children were institutionalized at an early age, they are now residing in their homes longer before this occurs (Petrowski et al., 2008; Wang and Brown, 2009). As a consequence, families and professional caregivers have different roles and responsibilities these days than before (Woodgate et al., 2015). For example, parents now have to simultaneously fulfill both the role of affectionate parent and healthcare provider. Also, parents play a vital role in the education and personal development of a child as they usually are the first and longest lasting carers (Jansen et al., 2017). It is increasingly recognised that parents of a child with a disability are the experts with regard to their own child and that they can provide professionals with valuable information (Kruithof et al., 2020). In addition, concerning societal changes, the realisation of a participation society places increasing demands on a family's informal network and social communities (Da Roit and De Klerk, 2014; Delsen, 2016). As a result, families of children with SPID face challenges when it comes to giving their child the best possible life and, at the same time, taking care of themselves as a family.

In order to provide optimal support to families who have a child with SPID, understanding how variables impact their family life is important. For example, research shows that the time demands of caring for these children negatively impacts family life while a family-centered approach, a way of working in partnership with families by professionals, is positively related to the FQoL (Luijkx et al., 2017; Vanderkerken et al., 2019). According to Zuna et al. (2010), FQoL should be considered as the outcome of multiple variables which interact with each other, and subsequently contributes to (new) family strengths, needs, and priorities. This should be considered as an ongoing process. Furthermore, it appears that the experiences and needs of a person with a disability and his or her caregivers is not static. There are changes over time when these families experience unique characteristics during various transitional phases, such as developing new expectations concerning the child with a disability and the parent role (DeMarle and Le Roux, 2001; Hastings, 2016). It may thus be beneficial to view variables related to FQoL from a longitudinal perspective.

Although several studies were conducted on variables that apply to families that have a child with an ID, the outcomes of these studies and their theoretical foundations seem to be inconclusive. Consequently, research into variables related to families that have a child with SPID is minimal. Therefore, the aim of this systematic review is to categorise variables related to the FQoL of families with a child with an ID, in order to gain a better understanding of variables related to the FQoL of those families. In this context, the following research questions will be examined:

1. Which variables are related to the FQoL of families with a child with (SP)ID?
2. To what extent do variables deviate for the different transitional phases that these families experience?
3. In what way is research into these variables based on existing theories pertaining to the FQoL?

## 2 Method

### 2.1 Design

A systematic review of the literature was carried out and reported using the Preferred Reporting Items for Systematic Reviews and Meta-Analysis (PRISMA) statement (Moher et al., 2015).

### 2.2 Search strategy

Five electronic databases were consulted: MedLine/PubMed, CINAHL, Embase, PsycINFO and Web of Science. This search was conducted in June 2019 and repeated in June 2020, with the assistance of an information specialist of the Hanzehogeschool Groningen. In an attempt to maximize the retrieval of relevant articles, a broad and sensitive search was conducted (see Appendix A). Search terms included both MeSH terms and free-text terms. Subsequently, a snowball and citation search method was used to identify additional relevant studies.

### 2.3 Selection criteria for studies

#### 2.3.1 Inclusion criteria

Publications were included when they met the following criteria:

• Published in a peer reviewed journal. Peer review is considered as a form of quality assessment (Kelly et al., 2014).
• Published in English between 2000 and 2020. The reason to include only English studies and to limit this review to recent literature is because the topic of this review is well presented in English-language publications and to better match current insights (Hempel et al., 2016).
• Studies evaluating variables related to the FQoL of families that have a child (0–30 years) with ID.
• Since conceptualisation of the FQoL is still under development, publications focusing on family functioning, family well-being, family adaptation, family impact, and family resilience were also included in the study. Similar studies show the same approach (e.g. Bhopti et al., 2016).

#### 2.3.2 Exclusion criteria

Publications were excluded for the following reasons:

• When the QoL was viewed only from the perspective of an individual family member rather than at the family level.
• If it was unclear whether the included child(ren) had an ID or not.
• Studies with a focus on family needs or demands were excluded because these concepts have their focus on requirements instead of quality of life.

### 2.4 Screening process

First, duplicates were eliminated in a selection process consisting of two phases. In the first phase, title and abstract were screened on the selection criteria. Subsequently, the publications that remained were reviewed full-text and assessed for eligibility. The selection process was performed by two persons. The first author (NL) and a second reviewer (FD) examined 10% of the obtained articles in the first phase. Disagreements were resolved by consensus discussion until the criteria of inclusion and exclusion were sufficiently clear. This resulted in an inter-rater reliability of 97%. The remaining titles were screened by one reviewer (FD). In the second phase two persons (NL and MS) independently and randomly screened all of the included articles full-text. Discrepancies were resolved with discussion between the two reviewers and, in the event of continued uncertainty, discussed with a third author (AW) until consensus was reached. This process of identifying eligible studies for this systematic review has also been applied in other studies (e.g. Willems et al., 2017).

### 2.5 Data extraction and synthesis

To summarize the data, a data extraction form was developed and included:

• General characteristics: first author and publication year, study purpose, sample including families (i.e. sample size and respondents) and child with ID (i.e. sample size, age range, ID level and percentage), country, method (i.e. design, measure), and quality score.
• Characteristics of the variables: relationship with the FQoL or another family concept (i.e. positive related, negative related, no relation, moderating effect).
• Characteristics of the used family theories: concept, framework.

After testing the form, the first author (NL) and a second reviewer (MS) independently performed 10% of the data extraction. The results were compared, and any disagreement was resolved with discussion between the researchers. If no consensus could be reached, a third author (AW) was consulted. Finally, the first author extracted all of the remaining data.

Subsequently, a narrative synthesis was conducted based on the FQoL theory described by Zuna et al. (2010). This unified theory for families of children with ID and other disabilities comprises four key concepts: (1) systemic concepts (i.e. systems, policies, and programs); (2) performance concepts (i.e. formal services, supports and practices); (3) individual family member concepts (i.e. demographics, characteristics, and beliefs); and (4) family unit concepts (i.e. family characteristics and family dynamics). These key concepts are interdependent and interrelated and collectively determine the FQoL (outcome). Variables identified within this review were categorised on the basis of these key concepts for the purpose of demonstrating their relationship with the FQoL. To determine which variables referred to families of children with SPID, they were classified by level of the ID: (1) mild; (2) moderate; and (3) severe or profound. Finally, variables were also categorised in the following three categories, i.e. (1) families with young children (0–8 years); (2) families with adolescents (9–18 years); and (3) launching children and moving on (19–30 years) to gain insight into their relation with transitional phases (Carter and McGoldrick, 1989).

### 2.6 Quality assessment

The selected articles were appraised using a critical review form for both quantitative (Law et al., 1998) and qualitative studies (Letts et al., 2007). Criteria were identified for the reviews (yes/no answers) with a maximum of 13 points for quantitative studies and up to 14 points for each qualitative study. For mixed method studies, a choice was made between these two options based on the focus of the research. First, two authors (NL and MS) assessed the quality of 10% of the publications independently. Subsequently, they compared their scores and agreement through consultation. This process was repeated until there was full consensus. Finally, the first author (NL) reviewed the remaining publications based on these agreements.

## 3 Results

**Figure 1:**
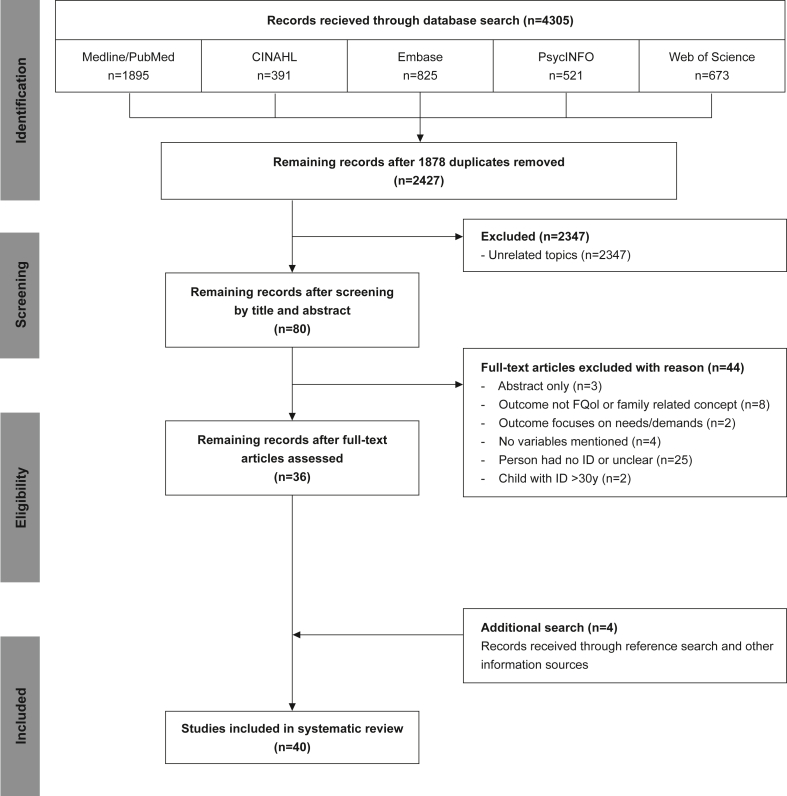
Flowchart for the selection of studies.

In this systematic review 2427 unique studies were identified (after removing duplicates) and, after applying inclusion and exclusion criteria, resulted in the inclusion of 40 full text studies (see Figure 1.).

### 3.1 General study characteristics

The included studies are summarized in Table 1.

**Table 1:** Overview of studies included.

Study 1st author; year	Study purpose	Sample 1) Families (n, respondents) 2) Children (n, age range, ID level, ID percentage) 3) Country	Method 1) Design 2) Measure	Family theory 1) Concept 2) Framework	Quality score Quantitative, max 13 Qualitative, max 14
Ajuwon, 2012	To analyse FQoL of families that have a child with ID and the relationship between families' life experiences and government policy and provision of services.	1) 80; main caregivers (mothers 98%) 2) 80; m = 12,3/sd = 7.85; NR; 100% 3) Nigeria	1) Mixed method; cross-sectional 2) Survey; FQOLS-2006	1) FQoL 2) FQoL framework	11/13
Balcells-Balcells, 2018	To determine the impact of supports and partnership on FQoL	1) 202; mothers (79%), fathers (18%), siblings (1%) 2) 202; 0–6; NR; NR 3) Spain	1) Quantitative; cross-sectional 2) Survey; BCFQOL	1) FQoL 2) Structural equation model	12/13
Bertelli, 2011	To study the correlation between QoL of adults with ID and relatives.	1) 27; mothers (56%), fathers (22%), siblings (22%) 2) 27; 27–50; NR; 100% 3) Italy	1) Mixed method; cross-sectional 2) Survey; FQoLS-2006	1) FQoL 2) QoL framework	12/13
Boehm et al., 2015	To examine QoL among families of transition-age adolescents with ID and/or autims.	1) 425; mothers (87%), fathers, (10%), grandparents (3%) 2) 425; 13–21; NR; 50% 3) USA	1) Quantitative; cross-sectional 3) Survey; BCFQOL	1) FQoL 2) FQoL framework	12/13
Boehm, 2019	To determine how parents of children with ID rate FQoL and what associations exist among FQOL and demographic factors, religiosity/spirituality, and relationships.	1) 529; mothers (87%), fathers (8%), siblings (2%), grandparents 2%) 2) 529; 0–74; NR; 100% 3) USA	1) Quantitative; cross-sectional 2) Survey; BCFQOL	1) FQoL 2) FQoL framework	10/13
Boelsma et al., 2018	To analyse factors that influence support from others and interaction with the community.	1) 25; parents (61%), siblings (26%), child with ID/DD (13%) 2) 7; 11–22; NR; 100% 3) Netherlands	1) Qualitative; cross-sectional 2) Interview	1) FQoL 2) FQoL framework	13/14
Brown et al., 2011	To investigate families' perceptions of family functioning during placement of their child with multiple diagnoses at residential facilities.	1) 25; parents 2) 23; 6–19; NR; 74% 3) UK	1) Qualitative; cross-sectional 2) Focusgroup, interview	1) FQoL 2) FQoL framework	9/14
Choi and Yoo, 2015	To identify resilience factors affecting adaptation of families with children with Down syndrome.	1) 125; mothers (94%), fathers 6%) 2) 126; 0–15, NR; 100% 3) Korea	1) Quantitative; cross-sectional 3) Survey; Family APGAR	1) Family adaptation 2) Patterson's conceptual framework of family resilience	9/13
Cohen et al., 2014	To examine the contribution to FQoL of family support beliefs, assistance from family members, and moderating effects of ethnicity and income.	1) 145; mothers (100%) 2) 145; 2–10; NR; 100% 3) USA	1) Quantitative; cross-sectional 2) Survey; BCFQOL	1) FQoL, Attitudinal familism 2) Attitudinal familism model	11/13
Davis and Gavida-Payne, 2009	To investigate the relationship between parental perceptions and experiences with family-centred support and FQOL.	1) 64; parents (mothers 95%) 2) 64; 3–5; NR; 6% 3) Australia	1) Quantitative; cross-sectional 2) Survey; BCFQOL	1) FQoL 2) Family systems theory	11/13
Foley et al., 2013	To explore relationships between FQoL, day occupations and activities of daily living of persons with Down syndrome.	1) 150; families 2) 150; 16–30; NR; 100% 3) Australia	1) Mixed method; cross-sectional 2) Survey; BCFQOL	1) FQoL, Family functioning 2) NR	10/13
Gardiner et al., 2018	To identify functional predictors of perceived impact of childhood disability among families of children with disabilities.	1) 216; mothers (82%) 2) 216; 4–13; NR; NR 3) Canada	1) Quantitative; cross-sectional 2) Survey; FICD	1) Family impact 2) NR	11/13
Hsiao (2014)	To examine family demands,social support and family functioning in families rearing children with Down syndrome.	1) 83; mothers (52%), fathers (48%) 2) 83; 4–17; NR; 100% 3) Taiwan	1) Quantitative; cross-sectional 2) Survey; FAD	1) Family functioning 2) NR	11/13
Hu et al., 2012	To explore the perceptions of QoL of families a child with ID.	1) 442; mothers (64%), fathers (31%), grandparents (3%) 2) 442; 0–18+; mild, moderate, severe; 100% 3) China	1) Quantitative; cross-sectional 2) Survey; BCFQOL	1) FQoL 2) FQoL framework	13/13
Ignjatovic et al., 2017	To examine the effects of newly introduced services on FQoL.	1) 153; mothers (68%), fathers (21%), grandparents (4%), foster family member (7%) 2) NR; 3–42; NR; 19% 3) Serbia	1) Quantitative; experimental longitudinal 2) Survey; BCFQOL	1) FQoL 2) FQoL framework	12/13
Isa et al., 2013	To determine the level of family impact in terms of overall impact, parent health related QoL and family functioning on families of children with disabilities.	1) 425; parents (96%), grandparents (3%), siblings, (1%) 2) 425; 2–18; NR; 73% 3) Malaysia	1) Quantitative; cross-sectional 2) Survey; PedsQL FIM	1) Family functioning, Family impact 2) NR	11/13
Lamb et al., 2016	To investigate factors related to family functioning and adaptation in caregivers of individuals with Rett syndrome.	1) 396; mothers (91%), fathers (8%) 2) 397; 1–50; NR; 100% 3) USA	1) Quantitative; cross-sectional 2) Survey; FAM-III	1) Family functioning 2) Thompson's transactional stress and coping model	12/13
Leonard e al., 2016	To analyse family experiences during transition to adulthood for children with ID.	1) 340; parents 2) 340; 15–29; NR; 100% 3) Australia	1) Mixed method; cross-sectional 2) Survey; Questionnaire	1) Family well-being 2) NR	9/14
Luijkx et al., 2017	To explore parents' appraisals of the impact of raising a child with profound intellectual and multiple disabilities on family life.	1) 56; mothers (66%), fathers (34%) 2) 56; 1–34; severe, profound; 100% 3) Netherlands	1) Quantitative; cross-sectional 2) Survey; FICD	1) Family impact 2) Family systems theory	11/13
Magill-Evans et al., 2001	To determine life experiences of families with and without a child having cerebral palsy during adolescence.	1) 162; mothers (39%), fathers (30%), siblings (31%) 2) 165; 13–15/19-23; NR; 25% 3) Canada	1) Quantitative; cross-sectional with control group 2) Survey; FAD	1) Family functioning 2) Ecological framework	12/13
Marchal et al., 2016	To determine if FQoL and family functioning of parents of children with Down syndrome differ from reference parents.	1) NR; mothers (65%), fathers (35%) 2) 88; 11–13; NR; NR 3) Netherlands	1) Mixed method; cross-sectional 2) Survey; Dutch Family Questionnaire	1) Family Functioning 2) NR	11/13
Mazaheri et al., 2013	To examine the effects of caring for a child with Prader–Willi syndrome on the mother and siblings.	1) 12: mothers (48%), siblings (52%) 2) 12; 1–27: NR; NR 3) USA	1) Mixed method; cross-sectional 2) Survey; PedsQL FIM	1) Family Functioning, Family Impact 2) NR	9/13
McConnell et al., 2014	To investigate resilience in families raising children with disabilities and behavior problems.	1) 538; mothers (88%), fathers (12%) 2) 538; 4–18; NR; 26% 3) Canada	1) Quantitative; cross-sectional 2) Survey; FAD	1) Family Functioning, Family Resilience, Family Adaptation, Family life congruence 2) Ecocultural theory	12/13
Mori et al., 2017	To investigate parental wellbeing and FQOl of families with the CDKL5 disorder.	1) 192; mothers (88%), fathers (11%) 2) 192; 0-34y; NR, 100% 3) Australia	1) Quantitative; cross-sectional 2) Survey; BCFQOL	1) FQoL 2) FQoL framework	10/13
Neikrug et al., 2011	To analyse the QoL of families raising a child with a disability.	1) 103; mothers (81%), fathers (4%) 2) 103; 1–31; NR; 9% 3) Israël	1) Mixed method; cross-sectional 2) Survey; FQOLS-2006	1) FQoL 2) FQOL framework	11/13
Povee et al., 2012	To explore factors that predict functioning in families with a child with Down syndrome.	1) 224; primary carers 2) 224; 4–25; NR; 100% 3) Australia	1) Mixed method; cross-sectional 2) Survey; FAD	1) Family Functioning 2) NR	12/13
Raspa et al., 2014	To examine adaptation across 7 dimensions of family life of families with a child with Fragile X syndrome.	1) 1099; mothers (89%) 2) 1394; 1–65; NR; NR 3) USA	1) Quantitative; cross-sectional 2) Survey	1) Family Adaptation, Family Empowerment, Family Life, FQoL 2) Conceptual model of family adaptation	11/13
Reilly et al., 2015	To analyse parent experiences and factors associated in four of the most common neurogenetic syndromes.	2) 381; mothers (89%) 1) 381; 4-19y; NR; NR 3) Ireland	1) Quantitative; cross-sectional 2) Survey	1) Family Functioning, Family Impact 2) NR	7/13
Rieger and McGrail, 2013	To investigate whether coping humor predicts of family functioning in parents of a child with disabilities.	1) 72: mother (82%), fathers (18%) 2) 72; 3–21; NR; NR 3) USA	1) Quantitative; cross-sectional 2) Survey; FACES IV	1) Family Functioning 2) Circumplex model	12/13
Rillotta et al., 2012	To investigate the FQOL of families having a member with intellectual/developmental disabilities.	1) 42; mothers (88%), fathers (2%); grantparent (2%), sibling (2%) 2) 42; 2–46; NR; NR 3) Australia	1) Mixed method; cross-sectional 2) Survey; FQOLS-2006	1) FQoL 2) NR	11/13
Rodrigues et al., 2018	To examine the impact of severe or profound ID on the FQoL of Brazilian families.	1) 15; mothers (100%) 2) 15; 5–24; severe, profound; 100% 3) Brazil	1) Qualitative; cross-sectional 2) Interview	1) FQoL 2) Family system theory	12/14
Scherz et al., 2016	To describe FQoL of families with a child with a severe disability.	1) 70; parents/legal guardians 2) 70; 0–18; mild, moderate, severe; 21% 3) Israël	1) Quantitative; cross-sectional 2) Survey; FQOLS-2006	1) FQoL 2) FQoL framework	11/13
Schippers and Van Boheemen, 2009	To explore and describe positive practices by partners in supporting young adults with ID.	1) 9; families 2) 9; 18–23; mild, moderate; 100% 3) Netherlands	1) Qualitative; longitudinal 2) Survey; Questionnaire, interview	1) FQoL 2) FQoL framework	5/14
Steel et al., 2011	To provide an in-depth analysis of the social and professional domains of FQol from the perspective of parents.	1) 25; mothers (96%), fathers (4%) 2) 27; 3–28; NR; 96% 3) Belgium	1) Mixed method; cross-sectional 2) Survey; FQOLS-2006, interview	1) FQoL 2) FQOL framework	10/13
Trute and Hiebert-Murthpy, 2002	To develop an instrument to assess the impact of a child with developmental disabilities on parents and family	1) 88; parents 2) 88; 5–12; NR; 29% 3) Canada	1) Quantitative; longitudinal 2) Survey; FICD	1) Family Functioning, Family Impact 2) Theory of primary appraisal	11/13
Vanderkerken et al., 2019	To investigate the relation between a family-centered approach and FQOL in families with a child with ID receiving home-based support.	1) 58; mothers (61%), fathers (39%) 2) 58; 1–19; mild, moderate, severe; 100% 3) Belgium	1) Quantitative; cross-sectional 2) Survey; BCFQOL	1) FQoL 2) FQoL framework	13/13
Vitale, 2015	To identify functioning of families with a child with Prader–Willi syndrome.	1) 20; mothers (75%), fathers (25%) 2) 20; 2–17; NR; 100% 3) USA	1) Qualitative; cross-sectional 2) Interviews	1) Family Functioning 2) NR	11/14
Wakimizu et al., 2011	To evaluate empowerment and related factors in families raising a child with developmental disabilities.	1) 225; mothers (97%) 2) 225; 5–18; NR; 6% 3) Japan	1) Quantitative; cross-sectional 2) Survey; FES	1) Family Empowerment 2) NR	12/13
Wang et al., 2004	To explore associations between family income and severity of disability and parents'satisfaction with FQOL	1) 280; parents (95%), 2) 280; 0–8; NR; 6% 3) USA	1) Quantitative; cross-sectional 2) Survey; BCFQOL	1) FQoL 2) FQoL framework	12/13
Wang et al., 2006	To test whether mothers and fathers similarly view FQOL embodied in one measure.	1) 107; parents (98%) 2) 107; 0–5; NR, 32% 3) USA	1) Quantitative; cross-sectional 2) Survey; BCFQOL	1) FQoL 2) FQoL framework	11/13

*Note.* n = total number; NR = not registrated; FQoL = family quality of life; QoL = quality of life; ID = intellectual disability.

The sample size per study ranged from 9 to 1099 participating families. In 32 studies (80%), the proportion of children with ID ranged from 6% to 100%. In the other eight studies, children with ID were included, however, their proportion remained unclear. A total of six (15%) of the 40 articles provided a full description of the levels of ID, five of which (13%) focused on SPID.

In 29 studies (73%), respondents were the primary caregiver or parents; in 11 studies (27%) also siblings, grandparents, or other caregivers were involved. The proportion of mothers as the respondent was highest in 29 studies and ranged between 39% and 100%. The studies were carried out in Africa (n = 1), Asia (n = 7), Europe (n = 11), America (n = 15), and Oceania (n = 6).

Of the 40 studies, 25 had a quantitative design, five were qualitative and ten used mixed methods. Most studies were cross-sectional (95%), and three were longitudinal of which one was also experimental. The most commonly used data collection method was a survey (n = 36); in six studies data collection (also) occurred through interviews and/or focus groups.

The quality scores of the quantitative studies ranged from 7 to 13 points (of a maximum of 13; *N = 34; M = 11; SD = 1.2*). Qualitive studies scored between 5 and 13 points (of a maximum of 14; *N = 6; M = 10; SD = 2,9*).

### 3.2 Variables related to the FQoL of families that have a child with ID

Table 2 shows variables related to the FQoL of families that have a child with an ID.

**Table 2:**
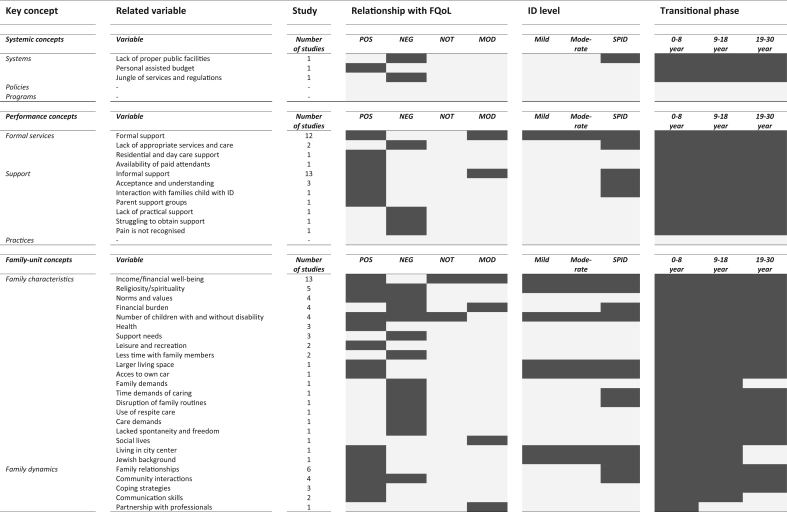

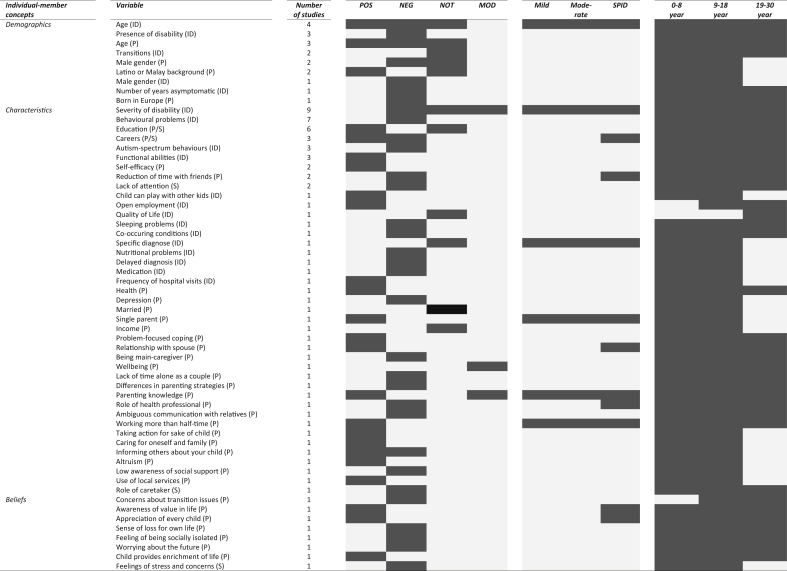
Variables related to the FQoL classified by key concept (Zuna et al., 2010), number of studies, relationship, ID level, and transitional phase.

*Note.* FQoL = family quality of life; ID = intellectual disability; POS = positive related; NEG = negative related; NOT = not related; MOD = moderator; SPID = severe or profound ID; P = parents; S = siblings.

The dark color indicates the type of relationship with FQoL; the level of ID of the children; or the age category of the children with an ID.

This review assessed a total of 98 variables, of which 58 were classified in individual-member concepts, 26 in family-unit concepts, 11 in performance concepts, and three in systemic concepts. Variables belonging to individual-member concepts related to both parents (n = 36), children with ID (n = 19), and siblings (n = 5). The two most frequently reported variables regarding children with ID were severity of a disability and behaviour problems (e.g. Boehm and Carter, 2019; Magill-Evans et al., 2001). In the case of parents and siblings, the two most common variables were education and careers (e.g. Neikrug et al., 2011; Rieger and McGrail, 2013). The three most mentioned variables in family-unit concepts were income/financial well-being, family relationships, and religiosity/spirituality (e.g. Boehm and Carter, 2019; Neikrug et al., 2011; Rodrigues et al., 2018). Formal and informal support were most common in performance concepts (e.g. Davis and Gavida-Payne, 2009; Neikrug et al., 2011). Systemic concepts listed three variables, i.e. lack of proper public facilities, personal assisted budget, and jungle of services and regulations (Rodrigues et al., 2018; Steel et al., 2011).

Variables were related positively, negatively, or both to the FQoL. Support was mentioned as being the most frequently found variable related positively to it (e.g. Boehm and Carter, 2019; Choi and Yoo, 2015). Severity of the disability and behavioural problems were most commonly found to be negatively related to it (e.g. Foley et al., 2013; Povee et al., 2012). Seven variables were ascertained both positively and negatively related to the FQoL, among them the most common being religiosity/spirituality and careers (e.g. Boehm et al., 2015; Neikrug et al., 2011). Variables could also be moderators, as is the case for support and income (e.g. Boehm and Carter, 2019; McConnel et al., 2014). Regarding six variables both a relationship and no relationship were demonstrated such as age, transitions, and severity of disability (Davis and Gavida-Payne, 2009; Hu et al., 2012; Hsiao, 2014; Magill-Evans et al., 2001).

### 3.3 Variables referring to families of a child with SPID

The level of ID was not described in 34 (85%) out of the 40 studies. As for the other studies, five focused on children with an SPID and referred to 30 variables. Of these 30 variables, 11 found themselves within individual-member concepts, 13 within family-unit concepts, five within performance concepts, and one within systemic concepts. The most frequently mentioned variables related to families of a child with SPID were support (n = 4), religiosity/spirituality (n = 3), finances (n = 3), and severity of disability (n = 2).

### 3.4 Differences according to transitional phases

Variables were divided into three transitional phases: 95 out of 98 variables were determined within the 0–8 year age group (families with young children); 96 were in the 9–18 year age group (families with adolescents); and 73 within the 19–30 year age group (launching children and moving on).

### 3.5 Theoretical foundations of FQoL

The FQoL concept was applied in 22 (55%) out of the 40 included studies. Another family concept was used in 18 studies, i.e. family functioning, family impact, family empowerment, family adaptation, family resilience, family well-being, family life and family system. In total, 26 studies referred to an underlying theoretical framework, however, with differences. To measure the FQoL, two quantitative instruments were utilized: BCFQOL scale (Beach Center on Disabilities, 2006) and FQOLS-2006 (Brown et al., 2006). Different instruments were used to measure the other family concepts.

## 4 Discussion

### 4.1 Main findings

The findings of this review indicate that studies were performed in different countries, and the sample size varied widely. In most cases, respondents were parents or the primary caregiver, usually mothers. The theoretical underpinnings of the studies that were included seemed to differ considerably. Most variables found concerned aspects of the individual and family, and only a smaller number referred to the environment (i.e. performance, system). Variables were either positively or negatively related to the FQoL and also interrelated. Results also showed contradictory findings, for example, variables that were related positively to it in one study and negatively related to it in another, or variables had both a relationship and no relationship with it. Besides, results did not provide a complete and consistent scenario concerning families of a child with SPID since they were rarely specifically described. Finally, knowlegde of the impact of transitional phases regarding the FQoL related variables is still minimal.

### 4.2 Theoretical reflections

Only a few variables were found at the systemic level, for example, that families of children with SPID struggle with a lack of proper public facilities and benefit from a flexible support system (Rodrigues et al., 2018; Steel et al., 2011). Though few systematic factors have been found, literature shows the key role of systemic variables within the conceptualisation of the FQOL (Zuna et al., 2009). Therefore the findings of the current review may be of extra interest.

Several studies demonstrated that families that have a child with a more severe disability have lower FQoL scores than families that have a child with less severe disabilities. However, if FQoL is affecting the (dis)abilities of the child or vice versa remains unclear. At the same time, results confirmed by this review showed that variables other than just the disability itself and the interrelatedness of variables determine the FQoL. For example, additional challenging behaviour of the child was realised as an important risk factor for disrupting family life (e.g. Davis and Gavida-Payne, 2009; Povee et al., 2012) while family-oriented support had a rather protective effect (e.g. Lamb et al., 2016; Steel et al., 2011). Research also demonstrated that variables, such as family resources and social supports, can moderate the impact of a disability on family life (Raspa et al., 2014). These outcomes are in accordance with the theoretical model of Zuna et al. (2010) in which the FQoL is considered as an outcome of a dynamic process consisting of multiple interactive factors. This study also shows that we still do not completely understand the relation between FQoL and the various related factors and therefore more research into this relationship is needed.

Conflicting results emerged regarding the impact of transitional phases. The classification of variables did not provide further information, and research aimed at ageing and transitions showed contradictory results. Nevertheless, previous studies have indicated characteristics of specific transitional stages. For example, ageing of people with SPID is accompanied with a reduction of social contacts and particularly after moving to a care facility (Hastings, 2016; Kamstra et al., 2015). It may be interesting to explore how this transition affects the FQoL and related variables. Therefore, despite inconclusive results it is still beneficial to view the FQoL related variables from a longitudinal and transitional perspective, and further research into this topic is necessary.

In most studies, the FQoL and other family concepts were measured only from the perspective of the primary caregiver or parents, usually mothers, therefore, there may be the risk of a one-sided focus with the results. However, research shows that mothers, fathers and siblings can all have their own and unique perspective on the disability and family life (Jansen et al., 2013; Wilder and Granlund, 2015) Understanding these differing views of family members in family oriented research is an important, but often overlooked, approach (McConnel et al., 2014). Therefore, this research can be considered as a basis for follow-up research in which different family members are involved.

### 4.3 Methodological reflections

A strength of this review is the broad and optimal search, which provided a substantial quantity of information. Synthesis of the results, including the use of a theoretical model, led to a structured overview of existing knowledge and identified some gaps in this area of research. Another strength of this review is the performance of a critical appraisal, showing that most studies can be categorised with a low risk of bias.

As with all research, some practical challenges were encountered when conducting this review. Variables related to families of children with an SPID should be interpreted with caution because they concern a small number of studies. The other studies were performed with families of children with less severe ID or the level of the ID was not distinguishable, therefore, it is questionable if these variables apply to families of children with SPID. Relevant articles could have been missed because only studies published from 2000 and written in English were included. However, in this review, the authors were mainly interested in the FQoL, and this concept has only actually received the attention of researchers for the past two decades. Moreover, a general shift towards the publication of studies in English may have diminished the risk of a language bias (Higgins and Green, 2011). Broadening the inclusion criteria for the target group would probably have yielded more information but also have resulted in a substantial number of studies and an even greater variety of research characteristics. Therefore, it was decided to refine the search to families that have a child with ID. A meta-analysis did not seem feasible due to the risk of heterogeneity of both samples, interventions, and outcome measures. Therefore, this review used narrative synthesis to analyse and report the findings. However, narrative synthesis has been criticised for its lack of transparency (Campbell et al., 2018). In order to address this criticism, the process of synthesis was clearly described.

### 4.4 Recommendations for future research

This review demonstrates that there is an urgent need for more knowledge into variables related to the FQoL of families that have a child with SPID. Future research should focus on variables that are particularly valid for these families from both a theoretically grounded, systemic, and longitudinal perspective while taking into account cultural diversity and all family member's unique and shared perspectives on the FQoL. It is preferred to combine different research methods as they can be mutually informative, such as qualitative studies that could help interpret findings from quantitative research (Bryman, 2012). In addition, there may be variables that are more easily amenable to change and, therefore, further exploration into dynamic and static variables can be beneficial in promoting the FQoL. Moreover, a follow-up to this research should not only look at variables presented in this review but also focus on other variables that may have the potential to affect familial relations. The instruments that are available today to measure FQoL are used primarily for evaluation purposes, for example, to determine the effect of an intervention or to compare the FQoL of different groups and factors related. In that way, we may not speak of ‘measure’ but more about ‘evaluate’. It may be interesting to investigate in follow-up studies if ‘sufficient’ FQoL should be measured. Based on this knowledge, recommendations for practice can be developed in order to support these families and to promote their FQoL, including their unique sociocultural context and environment.

## 5 Conclusion

This review provides insight into variables related to the FQoL of families with a child with ID. The results show various variables related to the FQoL and interrelated with each other. However, only a small number of studies have explored this topic in families of children with SPID. Moreover, there is still much uncertainty about how transitional phases may have an impact on FQoL related variables. Furthermore, there is variety in operationalising and examining the FQoL and related variables. Additional research is required to improve knowledge on variables related to the FQoL of families that have a child with SPID and to gain insight into how these variables may change over time.

## Declarations

### Author contribution statement

All authors listed have significantly contributed to the development and the writing of this article.

### Funding statement

This work was supported by the Netherlands Organisation for Health Research and Development, ZonMw (80-84500-98-355).

### Data availability statement

Data will be made available on request.

### Declaration of interests statement

The authors declare no conflict of interest.

### Additional information

No additional information is available for this paper.

## Appendix A Supplementary data

Luitwieler_et_al_2021_ Appendix A. Luitwieler_et_al_2021_ Appendix A.
