# Supplementary material for: Variables related to the quality of life of families that have a child with severe to profound intellectual disabilities: A systematic review
Source: Heliyon. 2021 Jun 24;7(7):e07372. doi: 10.1016/j.heliyon.2021.e07372 (PMC8353312; doi:10.1016/j.heliyon.2021.e07372)
Supplement: Luitwieler_et_al_2021_ Appendix A. [file mmc1.docx]

Appendix A.

(("Disabled Children"[Mesh] OR "Disabled Persons"[Mesh] OR "Mentally Disabled Persons"[Mesh] OR "Mental Disorders"[Mesh] OR "Intellectual Disability"[Mesh] OR "Cerebral Palsy"[Mesh] OR "Disabled child"[Title/Abstract] OR "Child with disabilities"[Title/Abstract] OR "Disabled children"[Title/Abstract] OR "Children with disabilities"[Title/Abstract] OR "Disabled people"[Title/Abstract] OR "People with disabilities"[Title/Abstract] OR "Disabled persons"[Title/Abstract] OR "Persons with disabilities"[Title/Abstract] OR "Mental disability"[Title/Abstract] OR "Mental disabilities"[Title/Abstract] OR "Mentally disabled"[Title/Abstract] OR "Mental disorder"[Title/Abstract] OR "Mental disorders"[Title/Abstract] OR "Mentally disordered"[Title/Abstract] OR "Mental disease"[Title/Abstract] OR "Mental diseases"[Title/Abstract] OR "Brain disorder"[Title/Abstract] OR "Brain disorders"[Title/Abstract] OR "Intellectual disability"[Title/Abstract] OR "Intellectual disabilities"[Title/Abstract] OR "Intellectually disabled"[Title/Abstract] OR "Intellectual impairment"[Title/Abstract] OR "Intellectually impaired"[Title/Abstract] OR "Intellectual development disorder"[Title/Abstract] OR "Intellectual development disorders"[Title/Abstract] OR "Intellectual and developmental disability"[Title/Abstract] OR "Intellectual and developmental disabilities"[Title/Abstract] OR "Cerebral Palsy"[Title/Abstract] OR "Handicapped child"[Title/Abstract] OR "Handicapped children"[Title/Abstract]) AND ("Family"[Mesh] OR "Family/psychology"[Mesh] OR "Family Relations"[Mesh] OR "Parent-Child Relations"[Mesh] OR "Father-Child Relations"[Mesh] OR "Mother-Child Relations"[Mesh] OR "Sibling Relations"[Mesh] OR "Family"[Title/Abstract] OR "Family relations"[Title/Abstract] OR "Family relationship"[Title/Abstract] OR "Family relationships"[Title/Abstract] OR "Family dynamics"[Title/Abstract] OR "Parent Child Relations"[Title/Abstract] OR "Parent Child Relationship"[Title/Abstract] OR "Parent Child Relationships"[Title/Abstract] OR "Parent-Child Relations"[Title/Abstract] OR "Parent-Child Relationship"[Title/Abstract] OR "Parent-Child Relationships"[Title/Abstract] OR "Father-Child Relations"[Title/Abstract] OR "Father-Child Relationship"[Title/Abstract] OR "Father-Child Relationships"[Title/Abstract] OR "Mother-Child Relations"[Title/Abstract] OR "Mother-Child Relationship"[Title/Abstract] OR "Mother-Child Relationships"[Title/Abstract] OR "Sibling relations"[Title/Abstract] OR "Sibling relationship"[Title/Abstract] OR "Sibling relationships"[Title/Abstract]) AND ("Quality of Life"[Mesh] OR "Quality of Life/psychology"[Mesh] OR "Family Health"[Mesh] OR "Activities of Daily Living"[Mesh] OR "Social Support"[Mesh] OR "Social Adjustment"[Mesh] OR "Adaptation, Psychological"[Mesh] OR "Quality of Life"[Title/Abstract] OR QoL[Title/Abstract] OR "Life quality"[Title/Abstract] OR "Family Quality of Life"[Title/Abstract] OR FQoL[Title/Abstract] OR "Health-Related Quality of Life"[Title/Abstract] OR HRQL[Title/Abstract] OR HRQoL[Title/Abstract] OR "Daily life activity"[Title/Abstract] OR "Activities of daily living"[Title/Abstract] OR "Family adaptation"[Title/Abstract] OR "Family health"[Title/Abstract] OR "Family Functioning"[Title/Abstract] OR "Family Life"[Title/Abstract] OR "Family well‐being"[Title/Abstract] OR "Family wellbeing"[Title/Abstract] OR "Social Support"[Title/Abstract] OR "Family Adjustment"[Title/Abstract] OR "Family satisfaction"[Title/Abstract]) AND ("Risk Factors"[Mesh] OR "Social Determinants of Health"[Mesh] OR "Family Health"[Mesh] OR "Determinant"[Title/Abstract] OR "Determinants"[Title/Abstract] OR "Influence"[Title/Abstract] OR "Influences"[Title/Abstract] OR "Influenced"[Title/Abstract] OR "Influencal"[Title/Abstract] OR "Factor"[Title/Abstract] OR "Factors"[Title/Abstract] OR "Cause"[Title/Abstract] OR "Causes"[Title/Abstract] OR "Caused"[Title/Abstract] OR "Causal"[Title/Abstract] OR "Risk"[Title/Abstract] OR "Risks"[Title/Abstract] OR "Indicator"[Title/Abstract] OR "Indicators"[Title/Abstract] OR "Indicate"[Title/Abstract] OR "Predictor"[Title/Abstract] OR "Predictors"[Title/Abstract] OR "Predict"[Title/Abstract] OR "Predictive"[Title/Abstract] OR "Predisposition"[Title/Abstract] OR "Predispose"[Title/Abstract] OR "Predisposed"[Title/Abstract] OR "Predisposing"[Title/Abstract] OR "Probability"[Title/Abstract] OR "Probably"[Title/Abstract] OR "Empowerment"[Title/Abstract] OR “Variable[Title/Abstract] OR Variables[Title/Abstract]))
